# Comment on Hack et al. Effect of Guarana (*Paullinia cupana*) on Cognitive Performance: A Systematic Review and Meta-Analysis. *Nutrients* 2023, *15*, 434

**DOI:** 10.3390/nu15082000

**Published:** 2023-04-21

**Authors:** Tom Gurney, Flaminia Ronca

**Affiliations:** Division of Surgery & Interventional Science, University College London, London W1T 7HA, UK; f.ronca@ucl.ac.uk

We have read the recent systematic review and meta-analysis by Hack et al. [1] entitled “Effect of Guarana (*Paullinia cupana*) on Cognitive Performance: A Systematic Review and Meta-Analysis” with great interest and we appreciate the authors’ contribution to this emerging field. We would like to draw the authors and reader attention to recently published data [2] which may support and strengthen their overall conclusions. Hack and colleagues raise several important points in their discussion and suggest future recommendations. Some of these have already been addressed in recent findings that were published before the submission of the systematic review and meta-analysis.

Hack and colleagues state that “*whether such performance changes are linked to the caffeine content or other bioavailable substances in guarana is unknown*” and suggest that “*future studies might investigate this further through matched-dose comparison trials between caffeine and guarana*”. Indeed, the study design and results of our recent investigation evaluated just that. Our paper was also in alignment with the suggestion that “*the caffeine content of guarana be expressed relative to body mass so that results become comparable in the future*”. The results of our study contribute to growing evidence that caffeine cannot act exclusively (following a matched 5 mg/kg caffeine dose to guarana supplementation) when positive effects on cognition are observed [2]. It is apparent that the combination of caffeine and other components contained within guarana, such as saponins, tannins, theobromine and theophylline, may provide superior simulant-like effects [2]. However, more research is certainly warranted. We concur with the authors that in their study “*ascertaining the added benefit above caffeine is not clearly seen in the studies included in the present analysis*” and hope that our findings in particular might further elucidate this gap in the field surrounding this topic area. 

We would also like to point the authors and reader to the effect of guarana supplementation on cognition in the context of exercise [2,3,4,5]. The wider applicability of guarana supplementation may be pertinent to activities that require bursts of high-intensity exercise accompanied by rapid decision making, including team sports, modern Pentathlon, law enforcement and military applications [2], aiding the interpretation and translation of the results into real-world practice. We thank the authors for their contribution to the field, and we concur with the observations made during the completion of their systematic and meta-analysis review. 
